# Integrated untargeted and targeted metabolomics combined with experimental validation reveal glutathione-related oxidative stress in pediatric IgA vasculitis nephritis

**DOI:** 10.3389/fphar.2026.1886454

**Published:** 2026-08-27

**Authors:** Leying Xi, Xiaoqi Xu, Qionqiong Xing, Shuang Xu, Hang Su, Yuying Sun, Jiale Liu, Xianqing Ren

**Affiliations:** 1 Pediatric Hospital of The First Affiliated Hospital of Henan University of Chinese Medicine, Zhengzhou, Henan, China; 2 School of Pediatrics, Henan University of Chinese Medicine, Zhengzhou, Henan, China; 3 The First Affiliated Hospital of Henan University of Chinese Medicine, Zhengzhou, Henan, China

**Keywords:** biomarker, glutathione, IgA vasculitis nephritis, lipid peroxidation, metabolomics, oxidative stress

## Abstract

**Objective:**

IgA vasculitis nephritis (IgAVN) is the principal cause of morbidity and mortality in children with IgA vasculitis (IgAV),the pathogenesis of the renal injury and predictive biomarkers are still unclear. In this study, we performed an integrated untargeted and targeted metabolomics to identify candidate biomarkers and explore oxidative stress-related alterations associated with IgAVN.

**Methods:**

Serum samples were collected from 115 IgAV and 111 IgAVN children. We conducted untargeted metabolomics for preliminary screening of potential biomarkers on 50 IgAV and 50 IgAVN children and then performed validation experiments based on targeted LC-MS/MS on 65 IgAV and 61 IgAVN children. In addition, an IgAVN animal model was established to preliminarily verify oxidative stress-related alterations associated with key metabolic abnormalities by assessing the levels of GSH, GSH/GSSG, MDA, 4-HNE, NRF2, HO-1, and GPX4.

**Results:**

A total of 45 differential metabolites were identified between the IgAV and IgAVN groups, including 23 upregulated and 22 downregulated metabolites, which were mainly enriched in glutathione metabolism, pyruvate metabolism, the citrate cycle, sphingolipid metabolism, and steroid hormone biosynthesis. Further targeted validation confirmed that six metabolites, including sphinganine, dehydroepiandrosterone, kynurenine, glutathione, nervonic acid, and creatine differed significantly between the two groups. The combined model based on these six metabolites showed good discriminatory performance, with an AUC of 0.903 and an area under the PRC curve of 0.909. In the animal experiments, the model group exhibited decreased GSH levels, a disrupted GSH/GSSG balance, increased MDA and 4-HNE levels, as well as abnormal expression of NRF2, HO-1, and GPX4.

**Conclusion:**

Children with IgAVN exhibit a distinct metabolic profile characterized by glutathione depletion, activation of inflammation-related tryptophan metabolism, lipid/membrane remodeling, and disordered energy metabolism. Sphinganine, dehydroepiandrosterone, kynurenine, glutathione, nervonic acid, and creatine may serve as potential biomarkers for IgAVN, and the combined model based on these metabolites showed good discriminatory ability and potential clinical utility. The consistency between the clinical metabolomics findings and the experimental evidence supports supports glutathione-related oxidative stress and the GSH-NRF2/HO-1/GPX4 axis as candidate pathways associated with IgAVN-related renal injury. These findings provide a basis for early risk identification and future mechanistic and pharmacological investigations in pediatric IgAVN.

## Introduction

IgA vasculitis (IgAV) is the most common systemic small vessel vasculitis in childhood, with an annual incidence of approximately 3–27 cases per 100,000 children (Watts et al., 2022). Renal involvement, referred to as IgA vasculitis nephritis (IgAVN), occurs in approximately 20%–80% of affected children, with the vast majority of cases being diagnosed within 6 months of disease onset (Parums, 2024; Chen and Mao, 2015). 1%–7% of children with IgAVN may progress to end-stage kidney disease, suggesting that the severity of renal involvement plays a decisive role in the long‐term prognosis of IgAV (Ozen et al., 2019). Recent IPNA clinical practice recommendations have highlighted that IgAVN is a highly heterogeneous renal disorder in childhood, and many issues regarding its diagnosis, risk stratification, and therapeutic management remain unresolved. In particular, early identification and precise assessment of renal involvement in children remain challenging (Vivarelli et al., 2025).

Renal biopsy is the gold standard for the diagnosis of IgAVN, which is an invasive procedure and is often difficult for parents to accept. In clinical practice, the diagnosis mainly relies on laboratory indicators, including urinalysis, 24-h urinary protein quantification, and the urinary protein-to-creatinine ratio. However, these indicators may lag behind early renal involvement and are insufficient for identifying children with IgAV who are at risk of developing nephritis (Zhang Y. et al., 2025). Therefore, the search for noninvasive, stable, and reproducible biomarkers that can reflect the underlying biological processes of the disease has become an important direction in IgAVN research. In recent years, omics studies especially metabolomics, which can directly capture changes in small-molecule metabolites under disease conditions, is considered to have great potential for the early diagnosis, prognostic evaluation, and mechanistic investigation of IgAVN (Zhou et al., 2025). Recent studies have suggested that patients with IgAVN exhibit metabolic features distinct from those of uncomplicated IgAV, and that some metabolites may have potential value in predicting IgAVN (Demir et al., 2020; Yu et al., 2024). However, the existing studies are generally limited by small sample sizes and remain largely at the stage of untargeted screening, with insufficient targeted validation and mechanistic support, thereby restricting their clinical translational value.

The pathophysiology of IgAVN and the mechanisms leading to nephritis are not well understood. An increasing evidence suggests that oxidative stress, lipid peroxidation, disordered energy metabolism, and abnormalities in ferroptosis-related defense systems are common features shared by multiple forms of IgA-associated renal injury, chronic kidney disease, and autoimmune diseases. Glutathione (GSH) and the related NRF2/HO-1/GPX4 axis play central roles in maintaining cellular redox homeostasis, suppressing lipid peroxidation, and limiting ferroptotic injury (Zhang L. et al., 2025; Qing et al., 2025; Lim et al., 2025). However, studies on glutathione-related oxidative stress in IgAVN remain very limited.

Therefore, this study aimed to integrate untargeted and targeted metabolomics to systematically characterize the serum metabolic profiles of children with IgAV and IgAVN, identify differential metabolites associated with renal involvement, and further validate their stability and potential diagnostic value through targeted quantification. In addition, animal experiments were conducted to preliminarily verify oxidative stress-related alterations associated with key metabolic abnormalities, with the aim of providing new metabolomics-based evidence for the early identification of pediatric IgAVN and for the investigation of pharmacologically relevant mechanisms.

## Materials and methods

### Clinical participants and sample preparation

IgAV patients and IgAVN patients were recruited from the First Affiliated Hospital of Henan University of Chinese Medicine between December 2023 and May 2024. Patients with IgAV fulfilled the EULAR/PRINTO/PRES Ankara 2008 classification criteria (Ozen et al., 2010). The IgAV group included patients who had no evidence of renal involvement at enrollment, including hematuria, proteinuria, or impaired renal function. IgAVN was defined as IgAV accompanied by renal involvement, including hematuria and/or proteinuria (Vivarelli et al., 2025). In the present cohort, all patients classified as having IgAVN underwent kidney biopsy and showed renal pathological findings compatible with IgAVN, including predominant glomerular IgA deposition. In addition, for patients in the IgAV group, we reviewed the follow-up records after sample collection. Children who remained free of renal involvement during the 2-month follow-up period were retained in the IgAV group, whereas those who subsequently developed IgAVN were excluded from the analysis. The study protocol conformed to the ethical guidelines of the current Declaration of Helsinki and received approval from the Ethics Committee of the Henan University of Chinese Medicine (2024HL-187–01). Written consent was obtained from all participants and their parents/guardians.

Blood samples were collected from IgAV and IgAVN patients, and before steroid or other immunosuppressive treatment was initiated. Blood samples were allowed to stand at room temperature for 1 h and then centrifuged at 3,000 × g at 4 °C for 10 min. The supernatant serum was collected, aliquoted, and stored at −80 °C until untargeted and targeted metabolomics.

### Untargeted liquid chromatography with tandem mass spectrometry analysis

An aliquot of 200 µL serum from each sample was transferred into a tube and mixed with 800 µL of pre-cooled methanol/acetonitrile solution (1:1, v/v). The mixture was vortexed for 60 s and incubated at −20 °C for 1 h to precipitate proteins. After centrifugation at 16,000 rpm at 4 °C for 20 min, the supernatant was collected and lyophilized, and the dried extracts were stored at −80 °C until measurement. Quality control (QC) samples were prepared by pooling 10 µL from each patient sample.

Samples were separated using an ultrahigh-performance liquid chromatography (UHPLC) system (Thermo Scientific™ Vanquish™ Flex) equipped with a C18 analytical column. The column temperature was maintained at 40 °C, and the flow rate was set to 0.4 mL/min. Mobile phase A consisted of water containing 0.1% formic acid, and mobile phase B consisted of acetonitrile containing 0.1% formic acid. The gradient elution program was as follows: 0–1 min, B increased linearly from 10% to 30%; 1–19 min, B increased linearly from 30% to 95%; 19–20 min, B was held at 95%; 20–20.1 min, B decreased linearly from 95% to 10%; and 20.1–20.5 min, B was held at 10%. During the entire analysis, samples were maintained at 4 °C in the autosampler. To improve analytical reliability, the injection order of all samples was randomized to minimize systematic bias, and one pooled QC sample was injected after every 10 study samples. Data were acquired in both positive and negative electrospray ionization (ESI) modes. After UHPLC separation, mass spectrometric detection was performed on a Thermo Scientific Q Exactive™ HF mass spectrometer.

The ESI source parameters were set as follows: sheath gas flow rate, 50 (arb units); auxiliary gas flow rate, 13 (arb units); sweep gas flow rate, 0 (arb units); capillary temperature, 300 °C; and spray voltage, +3.5 kV (positive mode) and −3.5 kV (negative mode). Full-scan MS was acquired over an m/z range of 67–1,000, and MS/MS (product ion) spectra were acquired over an m/z range of 67–1,000. Data-dependent MS/MS acquisition was performed in high-sensitivity mode using stepped normalized collision energies (NCE) of 15, 30, and 45.

Raw LC-MS/MS data from untargeted metabolomics were processed using Compound Discoverer 3.0 (Thermo Fisher Scientific, United States) for peak detection, retention time alignment, adduct merging, missing value imputation, background peak annotation, and metabolite identification. The processed data were further preprocessed in metaX for normalization, signal correction using locally estimated polynomial regression fitting, and removal of compounds with a coefficient of variation (CV) > 30% in QC samples. Metabolite annotation was performed based on accurate mass and MS/MS spectral matching against public databases. Multivariate analysis, including OPLS-DA, together with univariate analysis using Student’s t-test and fold change, was performed to identify candidate biomarkers. Candidate metabolites identified in the discovery stage were subsequently validated by targeted LC-MS/MS in MRM mode.

### Targeted liquid chromatography with tandem mass spectrometry verification of identified metabolites

Targeted liquid chromatography (LC) with tandem mass spectrometry (MS) analysis was performed using a UPLC-MS/MS system (ACQUITY UPLC-Xevo TQ-S). 9 standards were obtained from Sigma-Aldrich (St. Louis, MO, United States). All the standards were accurately weighed and prepared in an appropriate solution to obtain the individual stock solution at a concentration of 10 mmol/L. Aliquots of the individual stock solutions were then combined to prepare a mixed standard stock solution, in which the concentrations of the nine metabolites were 500, 125, 500, 500, 500, 0.5, 200, 500, and 500 μmol/L, respectively. The mixed standard stock solution was subsequently diluted stepwise with water to appropriate concentrations to prepare the working standard solutions.

Serum samples (200 µL) were extracted with 800 µL of prechilled methanol containing 100 μmol/L isotope-labeled internal standards, vortexed for 5 min, incubated at −80 °C for 8 h, and centrifuged at 16,000 × g for 15 min at 4 °C. The pellets were re-extracted with 200 µL of prechilled 80% methanol in water (v/v), sonicated for 5 min at 4 °C, vortexed for 5 min, and centrifuged under the same conditions. Combined supernatants were dried under vacuum at 4 °C, reconstituted in 100 µL of 20% methanol in water (v/v), and centrifuged at 18,000 × g for 15 min at 4 °C prior to LC-MS analysis.

Chromatographic separation was achieved on an InfinityLab Poroshell 120 HILIC-Z column (2.7 µm, 2.1 × 100 mm) at 30 °C with a flow rate of 0.4 mL/min and an injection volume of 2 µL. In positive ion mode, the mobile phases were acetonitrile and water, both containing 10 mM ammonium formate and 0.1% formic acid. The gradient was 0–2.0 min, 100%–90% A; 2.0–2.5 min, 90%–85% A; 2.5–4.0 min, 85%–60% A; 4.0–6.3 min, 60%–48% A; 6.3–6.4 min, 48%–100% A; and 6.4–7.5 min, 100% A. In negative ion mode, the mobile phases were acetonitrile and water containing 10 mM ammonium acetate and ammonia (pH 8.0), with the following gradient: 0–1.0 min, 80%–60% A; 1.0–3.5 min, 60%–50% A; 3.5–3.6 min, 50%–80% A; and 3.6–4.5 min, 80% A. Mass spectrometric detection was performed using electrospray ionization in positive and negative ion modes with multiple reaction monitoring. The source temperature was 500 °C, the ion spray voltage was 4,500/−4,500 V, curtain gas was 40 psi, nebulizer gas and auxiliary gas were 30 psi, and collision gas was set to medium.

Candidate biomarkers identified from the untargeted metabolomics analysis were further validated by targeted LC-MS/MS in MRM mode. The MRM transitions and compound-dependent parameters used for targeted metabolite quantification, including precursor ion, product ion, declustering potential (DP), collision energy (CE), and dwell time, are summarized in Supplementary Table S1. For analytes monitored with two transitions, one transition was used for quantification and the other for confirmation.

For targeted metabolomics, metabolites were quantified using calibration curves constructed from authentic standards, and the resulting quantitative data were analyzed using iMAP software (version 1.0; Metabo-Profile, Shanghai, China). Statistical significance for each metabolite was evaluated using Student’s t-test or the Wilcoxon rank-sum test.

### Animal experiments

Wistar male juvenile rats (n = 12; 4 weeks, 80–130 g) obtained from Charles River were housed under a 12 h day/night cycle at room temperature (22 °C) for the study [License No. SCXK (Zhejiang) 20200002]. The modeling approach was adapted and optimized from protocols previously established by our team (Xu et al., 2026). Because the etiology of human IgAVN remains incompletely understood, the present model was not intended to reproduce the initiating cause of the human disease. Instead, it was used as a phenotype-based experimental model to reproduce selected immunopathological and renal manifestations of IgAVN. Repeated immunization with OVA and Freund’s complete adjuvant was used to induce a sustained antigen-specific immune response, followed by repeated OVA challenges to promote systemic immune activation and immune-complex-associated tissue injury. Successful model establishment was evaluated by increased 24-h urinary protein excretion and urinary red blood cell counts, together with renal histopathological abnormalities and IgA deposition in renal tissue.

The model group (n = 6) was administered a decoction of heatnatured Chinese herbs (dried ginger, pepper, and long pepper) by gavage daily for 4 weeks to establish an internal environment of BloodHeat. Starting from the second week, an emulsion of ovalbumin (OVA) and Freund’s complete adjuvant was injected intraperitoneally once a week for 3 weeks. After the final intraperitoneal injection, an allergic reaction was induced by multiple tail vein and subcutaneous injections of OVA solution. The control group (n = 6) received an equivalent volume of purified water. Rats were anaesthetized by 1% sodium pentobarbital (40 mg/kg, i.p.). The serum and renal tissues were collected for western blotting and immunohistochemical analysis. The animal study was approved by the Animal Studies Ethics Committee of Henan University of Chinese Medicine (Approval No. IACUC-202408030).

### Statistical analysis

Statistical analyses were performed using GraphPad Prism 8. Quantitative data were summarized as mean ± standard deviation (SD) or median and interquartile range (IQR) [M (25th, 75th percentiles)] based on whether the data were normally distributed. Differences between groups were assessed using Student’s t-test or the Mann-Whitney U test. Serum metabolites with P < 0.05 were considered significantly different and were subjected to receiver operating characteristic (ROC) and related bioinformatic analyses. For the exploratory correlation analysis, Spearman correlation analysis was performed in IgAVN patients with available paired data to evaluate the associations between the six validated metabolites and renal injury-related parameters, including 24-h urinary protein and renal pathological grade. Renal pathological grade was analyzed as an ordered categorical variable according to the ISKDC classification. Spearman correlation coefficients and two-tailed P values were calculated.

## Results

### Clinical characteristics

A total of 226 participants were recruited. The baseline characteristics of the participants are shown in Table 1. 50 IgAV (31 females, 19 males, mean age 9.96 ± 3.22 years) and 50 IgAVN patients (24 females, 26 males, mean age 11.00 ± 3.149 years) for untargeted metabolomic analysis, there were no significant differences between age and gender (*P* = 0.09, 0.529 respectively). Another 65 IgAV (32 females, 33 males, mean age 10.95 ± 3.143 years) and 61 IgAVN (29 females, 32 males, mean age 11.20 ± 3.049 years) samples for targeted metabolomic analysis, there were no significant differences between age and gender (*P* = 0.828, 0.1064 respectively).

**TABLE 1:** Clinical characteristics of 115 IgAV patients and 111 IgAVN patients.

Characteristics	IgAV group (n = 115)	IgAVN group (n = 111)	*P*
Age (years)	10.50 ± 3.202	11.11 ± 3.082	0.1937
Sex (male/female)	1:1.211	1:0.914	0.3515
Renal pathology			
IIa (n, %)	—	14 (12.6%)	—
IIb (n, %)	—	2 (1.8%)	—
IIIa (n, %)	—	78 (70.3%)	—
IIIb (n, %)	—	15 (13.5%)	—
IV (n, %)	—	1 (0.9%)	—
V (n, %)	—	1 (0.9%)	—
VI (n, %)	—	0 (0)	—
Laboratory data			
24HUPr (g)	0.044 (0.031–0.065)	0.408 (0.160–1.179)	<0.0001
TP (g/L)	68.71 ± 4.847	65.57 ± 6.829	0.0095
ALB (g/L)	42.34 ± 2.570	40.43 ± 4.059	0.0061
BUN (mmol/L)	3.645 (3.108–4.285)	3.625 (3.275–4.113)	0.6175
Cr (umol/L)	33.65 (28.50–39.88)	41.40 (32.90–46.98)	0.7909
IgG (g/L)	10.70 (9.683–12.23)	9.365 (7.733–11.18)	0.0025
IgA (g/L)	2.110 (1.410–2.640)	2.090 (1.645–2.760)	0.5905
IgM (g/L)	1.185 (0.9225–1.615)	1.130 (0.8525–1.660)	0.6172
IgE (IU/ML)	44.40 (16.53–110.0)	43.40 (21.58–90.80)	0.8846
C3 (g/L)	0.960 (0.810–1.068)	0.900 (0.810–1.153)	0.06684
C4 (g/L)	0.1900 (0.1400–0.2425)	0.1750 (0.1400–0.2200)	0.5979
WBC (10^9^/L)	7.20 (5.10–10.28)	6.05 (4.875–8.050)	0.2616
PLT (10^9^/L)	279.5 ± 75.80	282.2 ± 87.21	0.9521

Renal histopathological findings were classified according to the International Study of Kidney Disease in Children (ISKDC) classification: I, minimal glomerular abnormalities; II, pure mesangial proliferation (MP); III, (a) focal or (b) diffuse MP with <50% crescents; IV, (a) focal or (b) diffuse MP, with 50%–75% crescents; V, (a) focal or (b) diffuse MP with >75% crescents; VI, membranoproliferative-like lesions.

As expected, the IgAVN group had significantly higher 24-h urinary protein levels than the IgAV group, whereas serum albumin and total protein levels were lower in the IgAVN group. No significant differences were observed in BUN or serum creatinine between the two groups.

### Untargeted metabolomic profiling of serum of IgAV and IgAVN patients

**FIGURE 1:**
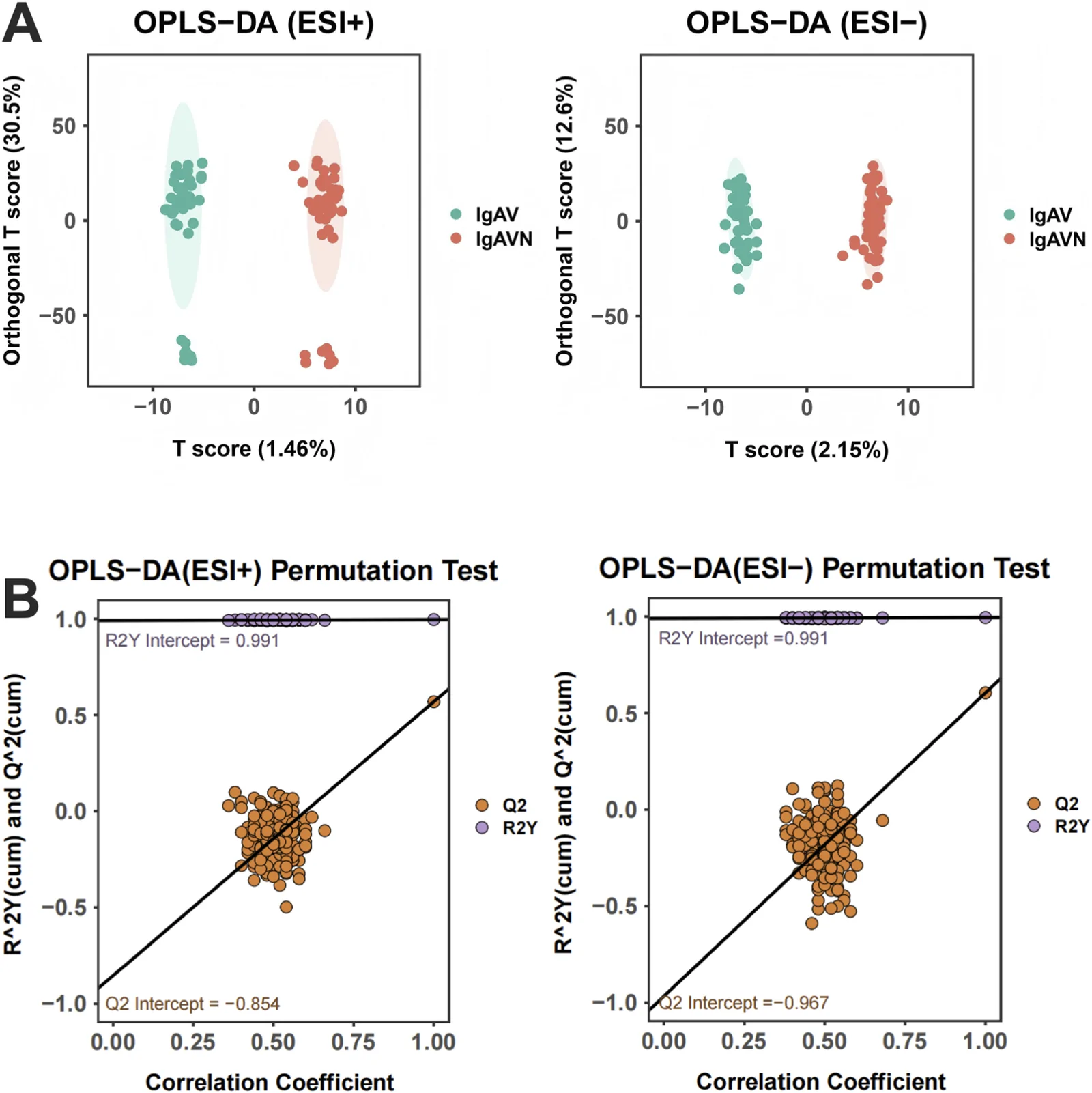
**(A)** OPLS-DA score plots of the IgAV and IgAVN groups in positive ion mode and negative ion mode. **(B)** Corresponding permutation tests in positive ion mode and negative ion mode.

Orthogonal partial least squares discriminant analysis (OPLS-DA), a supervised multivariate method that separates variation in the metabolite data into class-predictive and class-orthogonal components, was used to evaluate differences in the overall metabolic profiles between the IgAV and IgAVN groups. The OPLS-DA score plots showed a discernible separation trend between the two groups in both positive and negative ionization modes (Figure 1A). Following seven-fold cross-validation, the Q^2^ values were 0.567 and 0.603 for the positive and negative ionization modes, respectively. In addition, permutation testing yielded Q^2^ intercepts below zero in both modes (Figure 1B), suggesting that the models were not obviously overfitted and had acceptable predictive performance for exploratory analysis of group-related metabolic differences.

**FIGURE 2:**
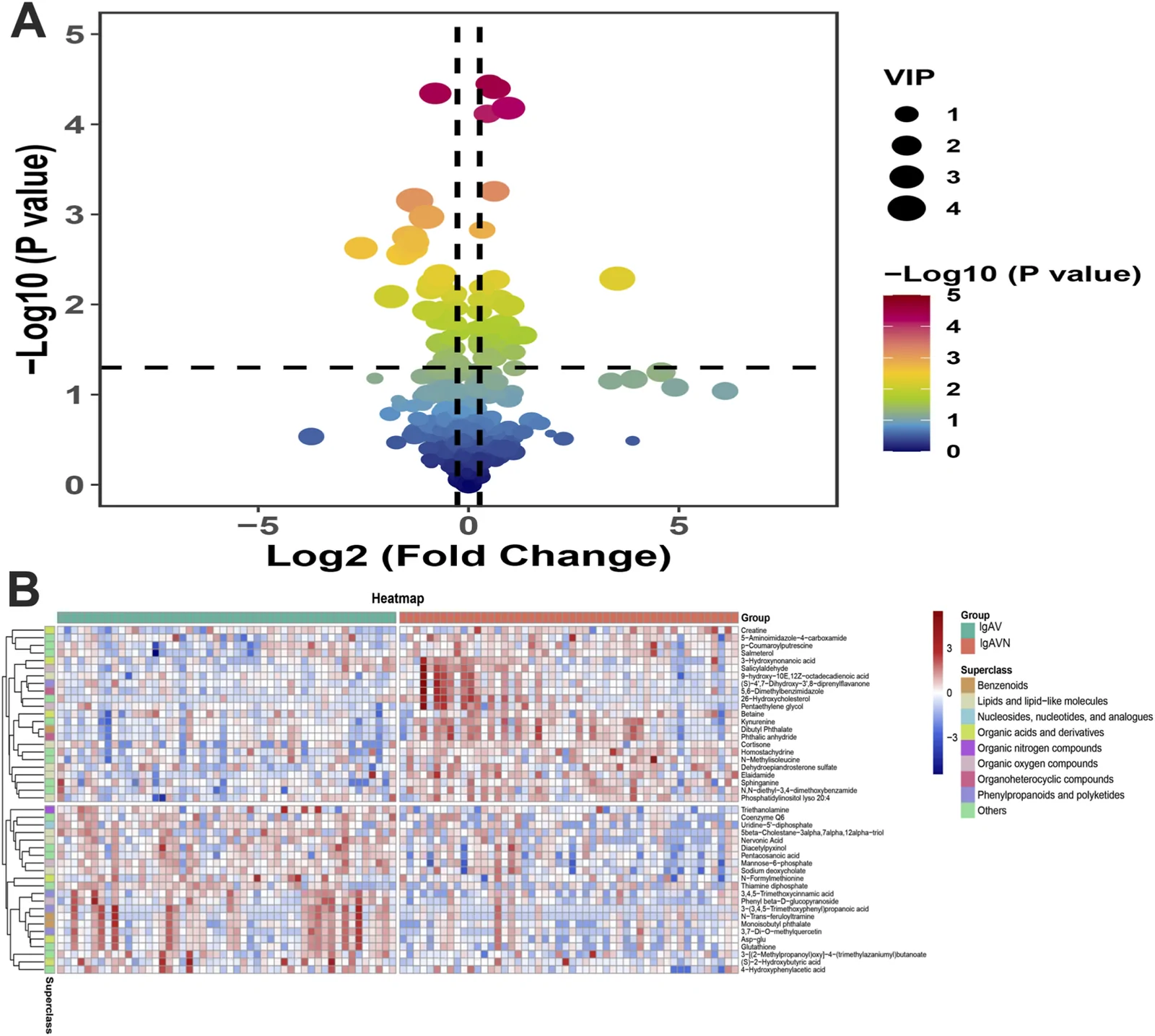
**(A)** Volcano plot of differential serum metabolites. **(B)** Hierarchical clustering heatmap of differential serum metabolites.

**FIGURE 3:**
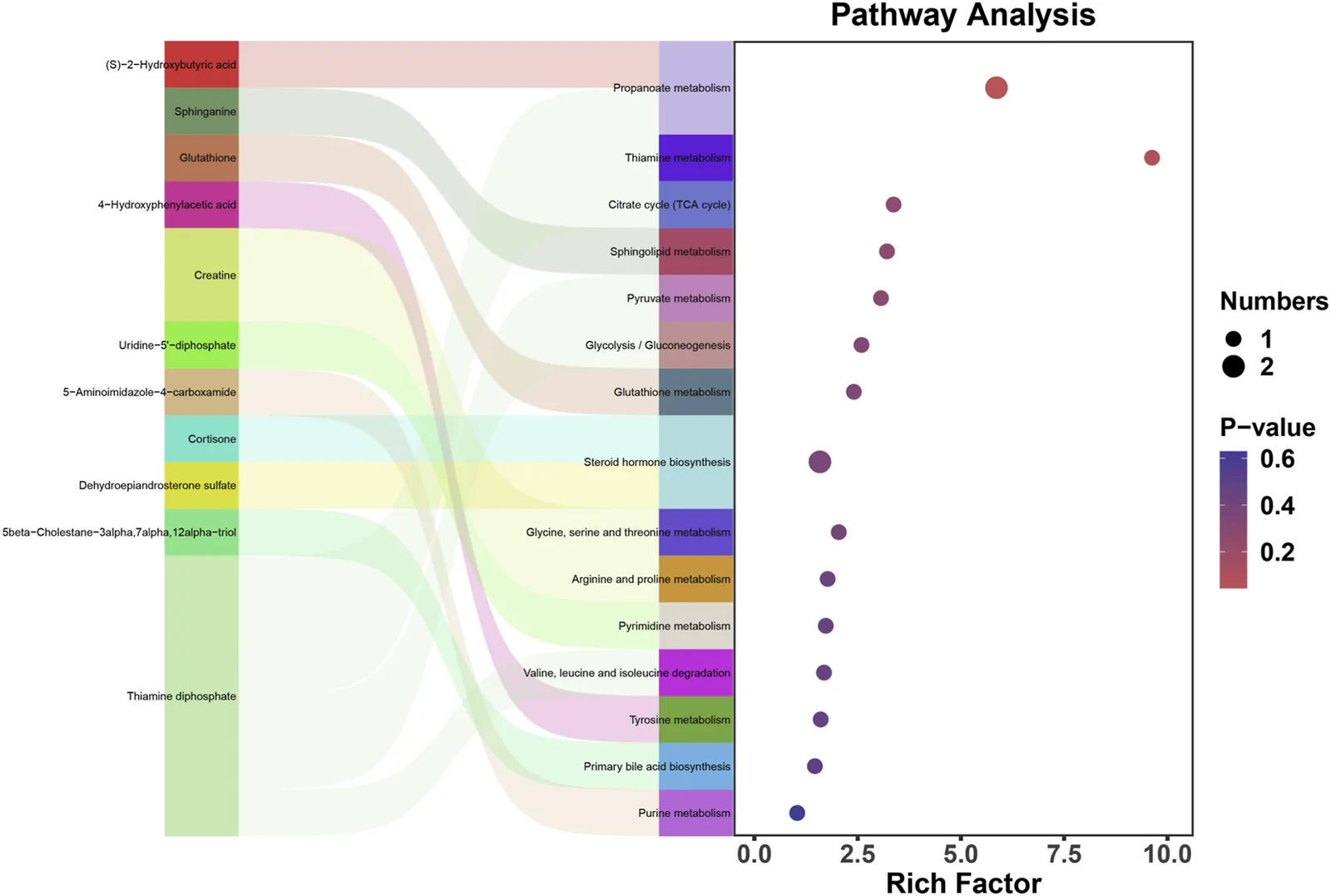
KEGG pathway analysis of differential metabolites.

Differential metabolites were screened using fold change analysis and univariate statistical analysis, and visualized using a volcano plot with VIP values represented by dot size (Figure 2A). Metabolites with |LogFC| > 1, P < 0.05, and VIP > 1 were defined as differential metabolites. A total of 45 differential metabolites were identified from the positive and negative ion mode datasets, including 23 upregulated and 22 downregulated metabolites. As shown in Figure 3, metabolites in the upper right quadrant were significantly upregulated, whereas those in the upper left quadrant were significantly downregulated, indicating marked metabolic differences between the IgAV and IgAVN group.

Hierarchical clustering analysis was performed on the 45 differential metabolites identified from the combined positive and negative ion mode datasets, and the results are shown in Figure 2B. The heatmap demonstrated a high degree of similarity in metabolite expression patterns within each group, whereas distinct differences were observed between the IgAV and IgAVN groups, indicating marked alterations in serum metabolic profiles. In addition, the differential metabolites were clustered into several groups with different expression patterns and were distributed across multiple chemical superclasses, including lipids and lipid-like molecules, organic acids and derivatives, and nucleosides, nucleotides, and analogues, suggesting that the metabolic disturbances between the two groups were multidimensional.

To further explore the biological relevance of the differential metabolites, KEGG pathway analysis was performed on the 45 metabolites identified between the IgAV and IgAVN group. The results showed that these metabolites were enriched in 15 metabolic pathways (Figure 3), mainly including propanoate metabolism, thiamine metabolism, the citrate cycle (TCA cycle), sphingolipid metabolism, pyruvate metabolism, glutathione metabolism, and steroid hormone biosynthesis. These findings suggest that the development of IgAVN may be associated with alterations in energy metabolism, lipid metabolism, redox homeostasis, and hormone-related metabolic processes.

To improve annotation confidence, differential features were further annotated by accurate mass and MS/MS spectral matching against public databases, resulting in 18 annotated differential compounds. Based on literature evidence, pathway relevance, biological significance, and feasibility for subsequent validation, nine compounds were selected as candidate biomarkers for targeted validation. As shown in Table 2, four candidate compounds, namely, Mannose-6-phosphate, Uridine 5′-diphosphate, Nervonic acid, and Glutathione, showed FC values below 1, indicating relative downregulation in the IgAVN group. In contrast, 5-Aminoimidazole-4-carboxamide, Creatine, Dehydroepiandrosterone, Sphinganine, and Kynurenine showed FC values above 1, indicating relative upregulation in the IgAVN group. These nine candidate compounds were subsequently subjected to targeted LC–MS/MS analysis for quantitative validation in clinical samples.

**TABLE 2:** Nine matched differential compounds.

Matched compound	HMDB id	FC	*P*
Mannose-6-phosphate	HMDB0001078	0.578	<0.001
Uridine-5′-diphosphate	HMDB0000295	0.624	0.005
Nervonic acid	HMDB0002368	0.499	0.001
Glutathione	HMDB0003337	0.381	0.001
5-Aminoimidazole-4-carboxamide	HMDB0003192	1.256	0.006
Creatine	HMDB0000064	1.939	0.01
Dehydroepiandrosterone	HMDB0000077	1.591	0.019
Sphinganine	HMDB0000269	1.363	0.039
Kynurenine	HMDB0000684	1.516	<0.001

### Targeted metabolomic analysis revealed alterative metabolites in IgAV and IgAVN patients

To enable subsequent targeted metabolomics validation, authentic standards of the nine candidate metabolites were analyzed by LC–MS/MS, and the corresponding MRM chromatograms were obtained (Supplementary Figure S1). The results showed that all candidate metabolites exhibited clear chromatographic peaks at their respective retention times, and the two MRM transitions for each metabolite showed consistent peak elution, indicating good qualitative consistency and method feasibility. Overall, the peak shapes and chromatographic separation were acceptable, supporting subsequent targeted quantification.

Subsequently, calibration curves were established using serial dilutions of the standard solutions. Linear regression analysis was performed using analyte concentration as the x-axis and instrument response as the y-axis. All nine candidate metabolites showed good linearity within their respective concentration ranges, with correlation coefficients greater than 0.99, indicating satisfactory linear range and quantitative performance of the established targeted LC–MS/MS method for subsequent sample analysis (Supplementary Table S2; Supplementary Figure S2)

**FIGURE 4:**
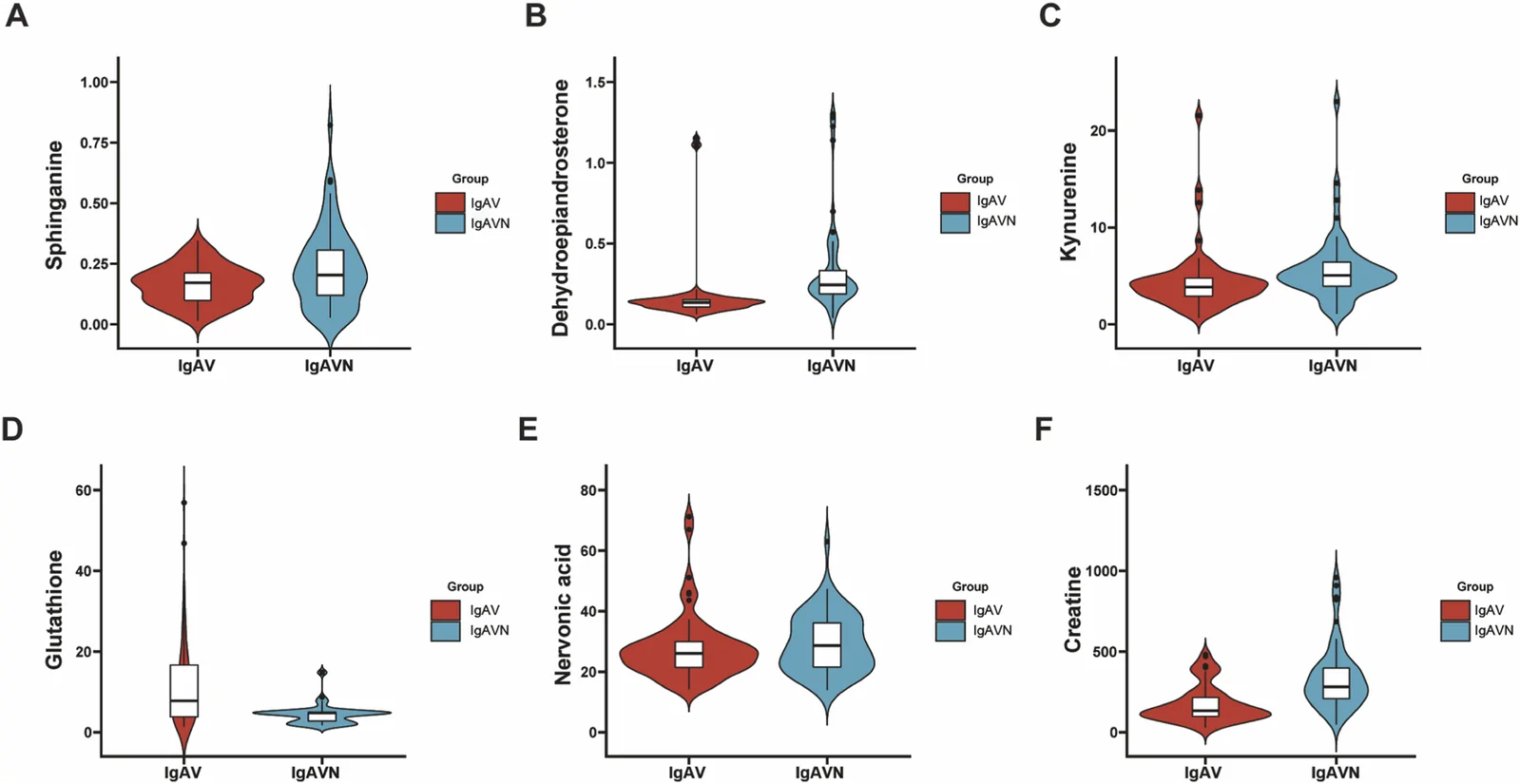
Distribution trends of six differential metabolites. **(A)** Sphinganine; **(B)** Dehydroepiandrosterone; **(C)** Kynurenine; **(D)** Glutathione; **(E)** Nervonic acid; **(F)** Creatine.

Based on the satisfactory chromatographic performance and linearity of the nine candidate metabolites, targeted LC–MS/MS quantification was further performed in clinical samples. Comparative analysis between the IgAV and IgAVN group showed that six of the nine candidate metabolites were significantly different between the two groups, including sphingosine, dehydroepiandrosterone, kynurenine, glutathione, nervonic acid, and creatine (Figure 4). Of these, sphingosine, dehydroepiandrosterone, kynurenine, nervonic acid, and creatine showed an upward trend in the IgAVN group, whereas glutathione showed a downward trend. These findings suggest that these metabolites may serve as potential biomarkers for renal injury in children with IgAV.

To further evaluate the combined discriminatory ability of the six validated differential metabolites, OPLS-DA and clustering analysis were performed (Supplementary Figure S3). The OPLS-DA model showed a certain degree of separation between the IgAV and IgAVN groups, suggesting that the six-metabolite panel had potential discriminatory value. Consistently, clustering analysis revealed different overall expression patterns between the two groups, indicating that this metabolite combination may have potential for group discrimination.

**FIGURE 5:**
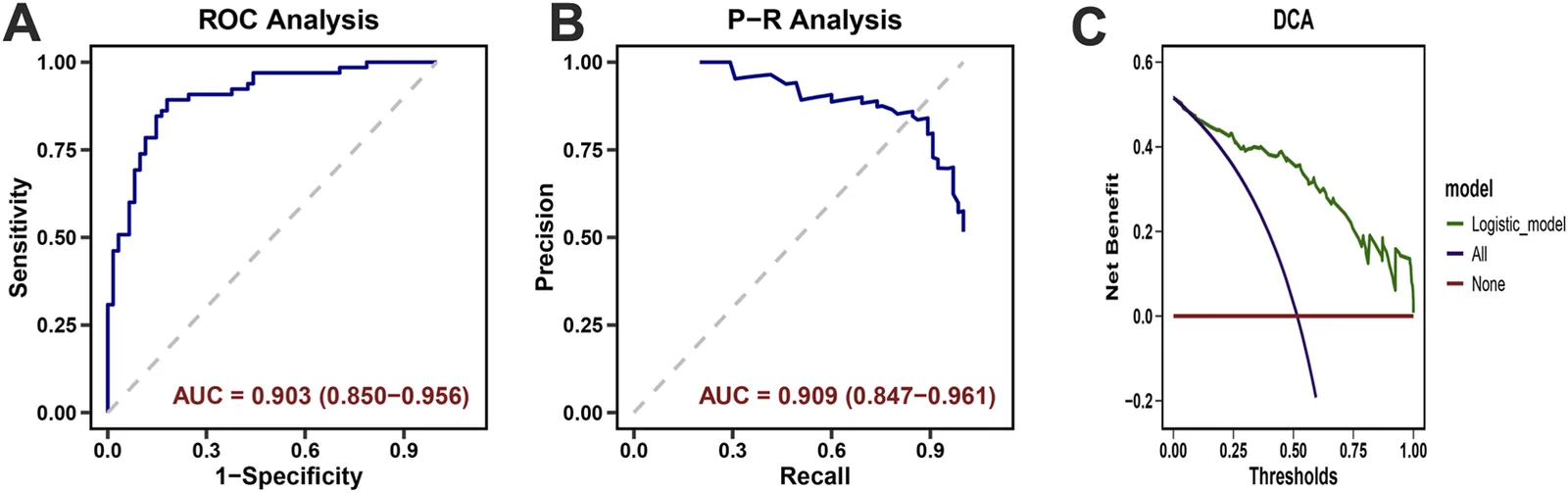
ROC **(A)**, PRC **(B)**, and DCA **(C)** analyses of the logistic regression model based on the six validated differential metabolites.

To further evaluate the discriminatory performance of the six-metabolite panel for IgAVN, a logistic regression model was constructed and assessed using ROC, PRC, and DCA analyses. As shown in Figure 5, the combined model achieved an AUC of 0.903 (95% CI: 0.850–0.956) in ROC analysis and 0.909 (95% CI: 0.847–0.961) in PRC analysis, indicating good discriminatory performance for distinguishing IgAV from IgAVN. DCA further showed that the model provided a higher net benefit than the “treat-all” or “treat-none” strategies within a certain threshold range, suggesting potential clinical utility.

**FIGURE 6:**
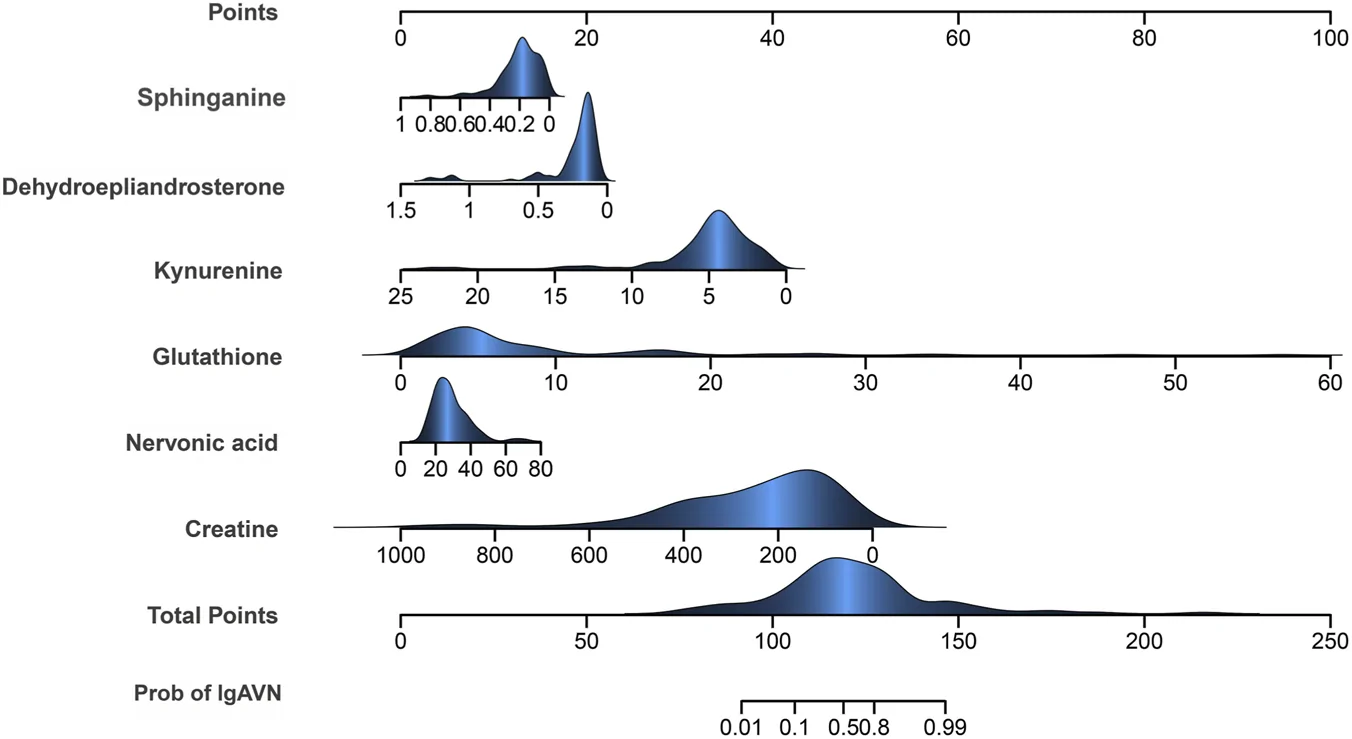
Nomogram for individualized prediction of IgAVN risk based on the six validated differential metabolites.

A nomogram was subsequently established based on the six validated differential metabolites for individualized risk assessment of IgAVN (Figure 6). The nomogram showed that the six-metabolite panel could be integrated into a visual scoring system for risk stratification, indicating its potential value in the assessment of renal involvement in children with IgAV.

In addition, spearman correlation analysis was further performed to evaluate the associations between 24-h urinary protein and the six validated metabolites in 61 IgAVN patients (Supplementary Figure S4). Glutathione showed a weak negative correlation with 24-h urinary protein (*r* = −0.3132, *P* = 0.014), whereas kynurenine showed a positive correlation trend that did not reach statistical significance (*r* = 0.2304, *P* = 0.074). Creatine, sphinganine, nervonic acid, and dehydroepiandrosterone were not significantly correlated with 24-h urinary protein. No significant correlations were observed between any of the six validated metabolites and renal pathological grade according to the ISKDC classification. These exploratory findings suggest that the six-metabolite panel may be more closely associated with the presence of renal involvement than with pathological severity in the current IgAVN cohort.

### Renal injury phenotype and oxidative stress–related alterations in the IgAVN animal model

**FIGURE 7:**
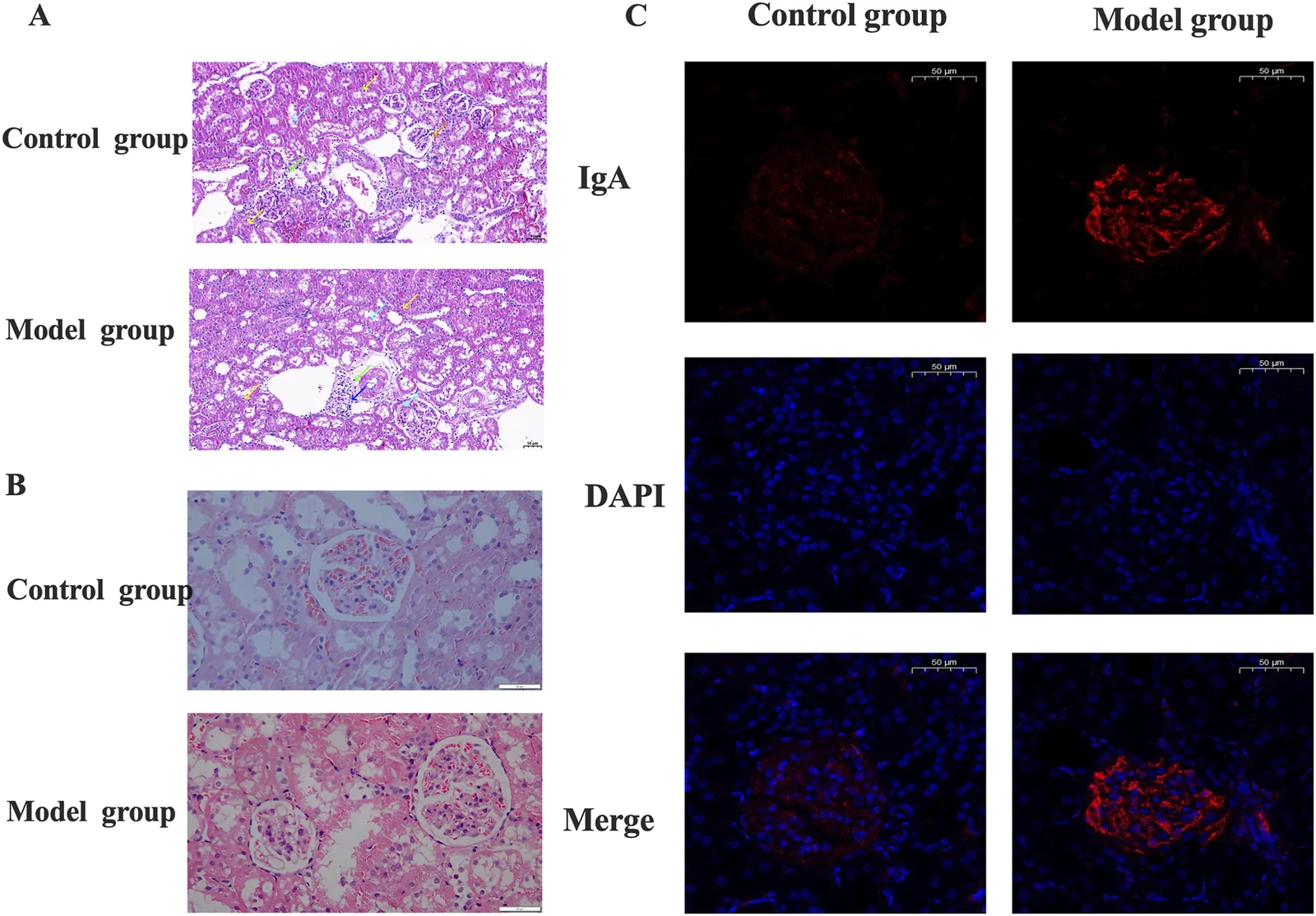
Pathological of renal tissues in the control and IgAVN groups. **(A)** HE staining of renal tissues in the control and IgAVN groups. **(B)** PAS staining of renal tissues in the control and IgAVN groups. **(C)** IgA immunofluorescence deposition in renal tissues of control and IgAVN groups.

**FIGURE 8:**
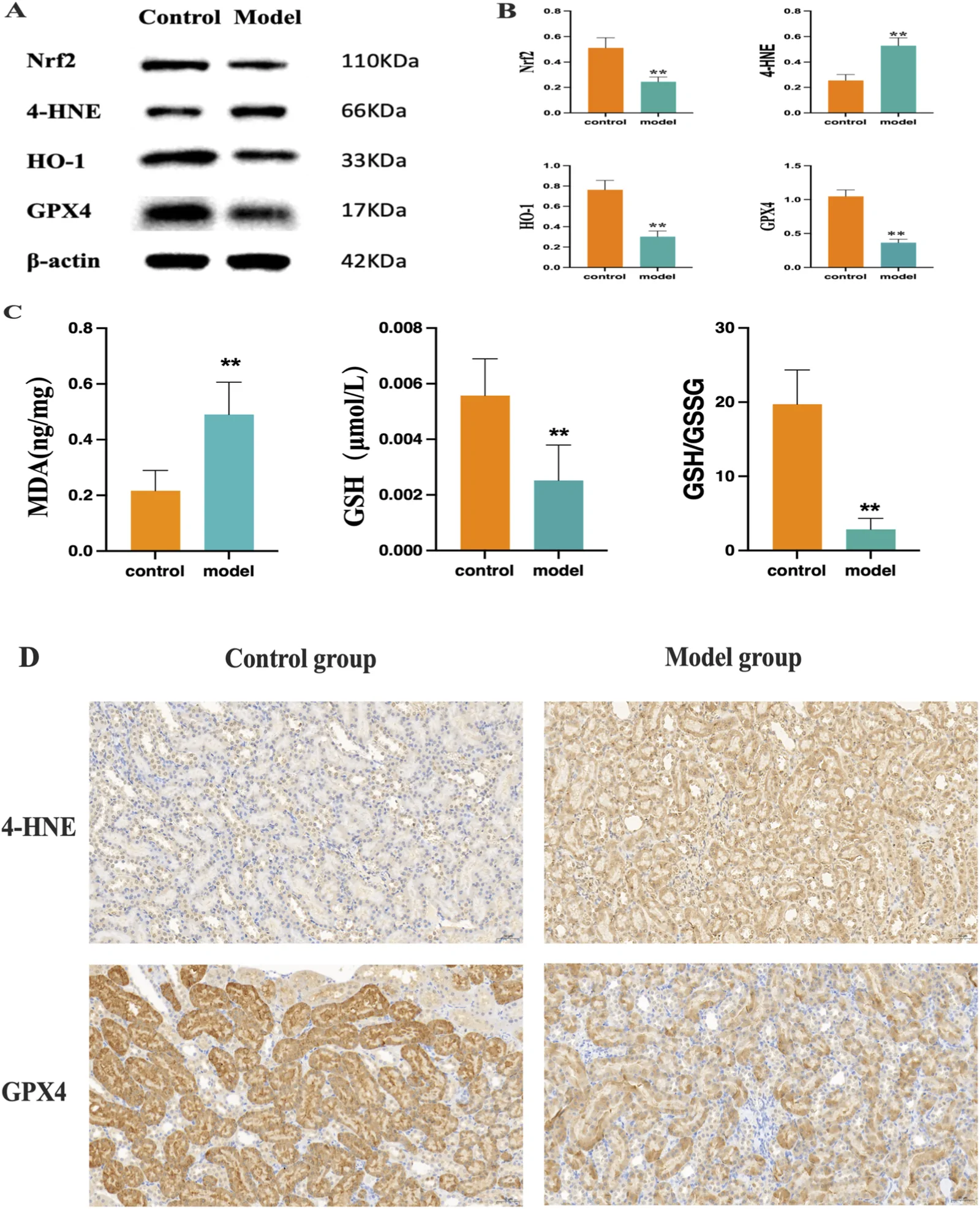
GSH/NRF2/HO-1/GPX4 pathway in renal tissues of IgAVN rats. **(A)** The band intensities of NRF2, HO-1, GPX4, and 4-HNE relative to β-actin. **(B)** Protein levels of NRF2, HO-1, GPX4, and 4-HNE in IgAVN rats. **(C)** The levels of MDA, GSH, and GSH/GSSG in IgAVN rats. **(D)** Representative images of renal tissue with immunohistochemical staining of 4-HNE and GPX4.

Given that both untargeted and targeted metabolomics analyses indicated glutathione-related metabolic abnormalities, this pathway was further examined in an IgAVN animal model. Compared with the control group, the model group exhibited significantly increased 24-h urinary protein (mg/24 h) (19.76 ± 5.53 vs. 11.02 ± 1.87, *P* = 0.01)) and red blood cell counts (/µL) (35.26 ± 12.37 vs. 21.02 ± 4.93, *P* = 0.024), and immunofluorescence analysis showed evident IgA deposition in renal tissues, indicating successful establishment of renal injury (Figure 7). In addition, the model group showed decreased GSH levels and a disrupted GSH/GSSG balance, accompanied by increased MDA and 4-HNE levels, suggesting enhanced oxidative stress and aggravated lipid peroxidation. Decreased of NRF2, HO-1, and GPX4 was also observed in renal tissues of the model group, indicating impairment of the antioxidant defense system (Figure 8). Immunohistochemical staining showed that, compared with the blank control group, 4-HNE-positive staining was markedly enhanced in renal tissues from the model group, mainly localized in the cytoplasm and membrane regions of renal tubular epithelial cells, indicating increased lipid peroxidation. In contrast, GPX4-positive staining was attenuated in the model group, suggesting reduced antioxidant defense against lipid peroxidation. Together, these findings were consistent with the clinical metabolomics results and support the involvement of glutathione depletion and oxidative stress in IgAVN-associated renal injury.

## Discussion

Using an integrated workflow of untargeted metabolomics, targeted validation, and animal verification, the present study systematically characterized the serum metabolic alterations associated with pediatric IgAVN. In addition to identifying six validated differential metabolites with potential biomarker value, our findings further linked these metabolomic changes to glutathione-related oxidative stress, thereby providing translational information and preliminary biological evidence related to renal injury in IgAVN. Our study found that the metabolic abnormalities in IgAVN were not sporadic changes in individual molecules, but rather reflected coordinated alterations across multiple pathways, including redox homeostasis, energy metabolism, lipid/membrane remodeling, and inflammation-related immunometabolism.

Among the six metabolites successfully validated by targeted analysis, the decrease in glutathione was of particular interest because it was observed in the clinical metabolomics analysis and was directionally consistent with oxidative stress-related alterations in the animal model. In this study, glutathione was decreased in the clinical metabolomics analysis, and this finding was further supported in the animal model, which showed reduced GSH levels, a disrupted GSH/GSSG ratio, increased MDA and 4-HNE levels, and abnormal NRF2/HO-1/GPX4 expression. Recent studies on renal oxidative stress have shown that GSH deficiency is closely associated with glomerular and tubular injury, amplification of inflammation, and chronic disease progression (Dong et al., 2023). Ferroptosis-related studies have clearly demonstrated that GSH depletion is a key upstream event leading to GPX4 dysfunction and the accumulation of lipid peroxides (Stockwell, 2022; Jiang et al., 2021; He et al., 2025). NRF2 is the central transcription factor governing the oxidative stress response, HO-1 is one of its classical downstream effector molecules, and GPX4 is the key enzyme that utilizes GSH to detoxify phospholipid peroxides and suppress ferroptosis. Recent studies have widely recognized the Nrf2–GSH–GPX4 axis as an essential defense system that limits lipid peroxidation and maintains renal cell survival. Dysregulation of this axis has been associated with aggravated injury in CKD, AKI, renal fibrosis, and immune-related kidney damage (Li et al., 2024). Furthermore, dysregulation of Nrf2/GSH/GPX4 may also contribute to the pathogenesis of autoimmune diseases, including systemic lupus erythematosus, rheumatoid arthritis, and psoriasis, by modulating immune cell ferroptosis, the inflammatory microenvironment, and lipid peroxidation (He et al., 2025; Liu et al., 2025). Notably, a 2025 study suggested that altered GPX4 expression in proximal tubular cells is associated with oxidative stress and ferroptosis in IgA nephropathy. Although IgAVN and IgA nephropathy are not completely identical, they share characteristics within the spectrum of IgA-associated renal injury (Qing et al., 2025). Therefore, our findings not only support the involvement of oxidative stress in IgAVN, but also further suggest that the GSH–NRF2/HO-1/GPX4 axis may represent a candidate pathway for further mechanistic validation for further investigation in IgAVN as well as other autoimmune diseases. However, the tissue localization of these oxidative stress-related changes requires cautious interpretation.

In the animal model, immunohistochemical staining showed that enhanced 4-HNE positivity and reduced GPX4 expression were mainly localized to the renal tubular epithelial compartment. This finding should be interpreted cautiously. Renal tubular epithelial cells are metabolically active and are particularly susceptible to oxidative stress and lipid peroxidation. In IgAVN, glomerular immune injury, proteinuria, inflammatory mediator release, and microcirculatory abnormalities may all contribute to secondary tubular oxidative stress. Therefore, the tubular staining pattern observed in this study may reflect one component of renal injury, but it does not establish the GSH-NRF2/HO-1/GPX4 axis as an IgAVN-specific or primary tubular mechanism. Similar oxidative stress-related responses may also occur as downstream injury processes in other glomerular diseases. Further validation using human renal tissue, cell-type-specific markers, and compartment-specific analyses of glomerular and tubular regions will be required.

Kynurenine is an important metabolite produced from tryptophan through the IDO/TDO pathway, and its elevation is commonly regarded as a marker of inflammation-driven immune-metabolic remodeling. Studies in kidney diseases have suggested that abnormalities in the kynurenine pathway are associated with inflammatory activity, endothelial injury, and deterioration of renal function (Zakrocka and Zaluska, 2022). In the present study, kynurenine was elevated in the IgAVN group, suggesting that, compared with uncomplicated IgAV, children with IgAVN may exhibit a more pronounced inflammatory drive and immune-metabolic disturbance.

Sphingolipids are not only structural components of the cell membrane but also active signaling molecules involved in the regulation of apoptosis, inflammation, migration, and stress responses. A 2024 review entitled Sphingolipids and Chronic Kidney Disease highlighted that ceramides, sphinganine, and their phosphorylated forms are closely associated with glomerular barrier function, tubular inflammation, and CKD progression (Sakic et al., 2024). In addition, a 2023 serum metabolomics study in IgA nephropathy reported significant abnormalities in sphingolipid and lipid metabolism (Dong et al., 2023). In the present study, elevated sphingosine suggested that alterations in membrane lipid composition and signaling lipids may occur in IgAVN. The increase in nervonic acid further reinforced the conclusion of disturbed lipid metabolism, as changes in nervonic acid are often regarded as an indirect reflection of altered membrane lipid homeostasis and fatty acid metabolic status. Taken together, the enrichment of sphingolipid metabolism, elevated sphingosine, and increased nervonic acid suggest that lipid reprogramming may represent a common and important direction for continued investigation in IgA-associated renal injury. Given that the fundamental pathological background of IgAVN includes small-vessel vasculitis and glomerular involvement, such sphingolipid abnormalities may be related to endothelial injury and activation of inflammatory signaling.

Changes in creatine levels generally suggest a reorganization of high-energy phosphate metabolism or a compensatory metabolic state within tissues. In the present study, KEGG pathway analysis indicated abnormalities in pyruvate metabolism, propanoate metabolism, and the citrate cycle, which together with the observed change in creatine constitute a coherent line of evidence for energy metabolic remodeling. In recent years, kidney disease metabolomics has increasingly emphasized that alterations in mitochondrial energy production, fatty acid oxidation, and substrate utilization represent common mechanisms underlying the transition from acute injury to chronic progression (Lumpuy-Castillo et al., 2024). One study included 662 biopsy-confirmed patients with IgAN and used machine learning to predict prognosis in patients with glomerulosclerosis <25%, the results showed that serum creatine, eGFR, serum triglycerides, crescent proportion, proteinuria, and complement C3 were strong predictors of IgAN prognosis (Lin et al., 2022). Another large retrospective study involving 1,165 biopsy-confirmed patients with IgAN found that, compared with the non-nephrotic-range proteinuria group, patients with IgAN and nephrotic-range proteinuria had higher serum creatine levels, lower eGFR and uric acid levels, more acute lesions, and poorer long-term clinical outcomes (Han et al., 2019).

DHEA and its sulfated form, DHEAS, are important nodes within the steroid hormone metabolic network and are closely linked to immune regulation, stress responses, inflammatory control, and metabolic status. Previous studies have shown that DHEA/DHEAS levels are associated with the overall condition and clinical outcomes of patients with CKD, although their directional significance in specific renal pathologies remains unclear. Recent reviews on DHEA have focused more on its broad pharmacological effects and its neuro-endocrine-immune regulatory functions rather than kidney disease–specific mechanisms (Nenezic et al., 2023). Studies on the relationship between DHEA and renal injury are relatively limited and have mainly focused on oxidative damage. Current evidence suggests that DHEA can reverse the inhibitory effect of high-glucose conditions on the growth of glomerular mesangial cells and alleviate oxidative injury by maintaining intracellular reduced glutathione levels and membrane Na+/K+ ATPase activity (Brignardello et al., 2000). Other studies have also shown that DHEA can attenuate the oxidative stress associated with ischemia/reperfusion-induced renal injury, reduce NOS, iNOS, and TNF-α levels, alleviate proximal tubular damage, and exert a protective effect on the kidney (Aragno et al., 2003). In a rat model of renal ischemia–reperfusion, DHEA treatment improved survival, renal function, and renal structure, as well as the inflammatory response following sublethal ischemia–reperfusion injury (Hosszu et al., 2017). Our findings further suggest that steroid hormone–related metabolism may be involved in the pathophysiology of IgAVN; however, whether this alteration reflects a compensatory response, a stress-related change, or part of the pathogenic process itself remains to be clarified.

From the perspective of study design, one of the major strengths of the present work lies in the implementation of a complete workflow consisting of untargeted discovery, targeted validation, and integrated modeling. From the perspective of translation, the combined model based on the six metabolites showed good discriminatory performance in the present cohort, distinguishing IgAVN from IgAVN. This suggests that the six-metabolite panel may have potential value as a candidate metabolic signature associated with renal involvement in children with IgAV. However, this model has not yet been validated in an external cohort, and the present study did not include sufficient longitudinal samples. Therefore, it remains unclear whether these metabolites can predict delayed-onset nephritis, monitor treatment response, or reflect long-term renal outcomes. Future multicenter studies with prospective follow-up are therefore needed to further evaluate the stability of this metabolite panel. Among these six molecules, glutathione is particularly noteworthy because it is supported by clinical metabolomic differences, experimental validation in the animal model, and mechanistic evidence, making it the most promising candidate for further development as a key link between metabolic biomarkers and disease pathophysiology.

The changes in glutathione metabolism and lipid peroxidation observed in this study also suggest a possible pharmacological implication. In kidney injury research, N-acetylcysteine and other glutathione-restoring strategies have been reported to attenuate oxidative damage (Hernandez-Cruz et al., 2024), while ferroptosis inhibitors and NRF2-activating approaches can reduce lipid peroxidation and preserve renal cell viability (Zhao et al., 2023; Bondi et al., 2024). These findings suggest that the GSH-NRF2/HO-1/GPX4 pathway may be worth further investigation in IgAVN. However, direct evidence in pediatric IgAVN is still lacking. Most available data come from animal or cell models, and the efficacy, optimal timing, and safety of such interventions in children remain unclear. Therefore, our findings provide a rationale for future pharmacological studies.

This study still has several limitations. First, the clinical sample size was relatively small and derived from a single center, therefore, larger cohorts and multicenter studies are still needed to validate the stability of these six metabolites. Second, the study was cross-sectional and did not include sufficient paired pre- and post-treatment serum samples, limiting our ability to evaluate whether these metabolites can monitor treatment response or predict individual outcomes. Third, validation in human renal biopsy tissue was not performed. In addition, the animal experiments currently mainly support the glutathione/oxidative stress axis, whereas the specific mechanisms underlying the other five differential metabolites remain to be directly validated. Finally, although the animal data suggest that GSH depletion, enhanced lipid peroxidation, and abnormal NRF2/HO-1/GPX4 expression coexist with renal injury, a causal relationship has not yet been established through NAC or other pharmacological interventions. Future studies should further investigate the GSH–NRF2/HO-1/GPX4 axis using interventional approaches and evaluate the six-metabolite model in external validation cohorts.

## Conclusion

In summary, through a workflow encompassing untargeted screening, targeted validation, and experimental verification, this study showed that children with IgAVN exhibit a distinct serum metabolic profile characterized by glutathione depletion, activation of inflammation-related tryptophan metabolism, and lipid and energy metabolic remodeling. Six differential metabolites, including sphinganine, dehydroepiandrosterone, kynurenine, glutathione, nervonic acid, and creatine, were further validated, and the combined model based on these metabolites showed good discriminatory performance in distinguishing IgAVN from uncomplicated IgAV in the present cohort. Moreover, the clinical metabolomics findings were directionally consistent with the IgAVN-like animal model results, which showed decreased GSH levels, disrupted GSH/GSSG balance, increased MDA and 4-HNE levels, and altered expression of NRF2, HO-1, and GPX4. These findings support glutathione-related oxidative stress and the GSH-NRF2/HO-1/GPX4 axis as candidate pathways associated with IgAVN-related renal injury. Further studies using human renal tissue validation, longitudinal clinical cohorts, and pharmacological intervention experiments are needed to confirm their tissue-level relevance, clinical utility, and mechanistic role in pediatric IgAVN.

## Data Availability

The original contributions presented in the study are included in the article/Supplementary Material, further inquiries can be directed to the corresponding author.
