# Supplementary material for: Integrated untargeted and targeted metabolomics combined with experimental validation reveal glutathione-related oxidative stress in pediatric IgA vasculitis nephritis
Source: Front Pharmacol. 2026 Aug 27;17:1886454. doi: 10.3389/fphar.2026.1886454 (PMC13557959; doi:10.3389/fphar.2026.1886454)
Supplement: Supplementary file 1 [file Table1.docx]

Supplementary Table 1 Multiple reaction monitoring parameters for the nine candidate metabolites.

| No. | Precursor ion | Product ion | Dwell time（ms） | Analyte | DP | CE |
| --- | --- | --- | --- | --- | --- | --- |
| 1 | 271.2 | 81.1 | 40 | Dehydroepiandrosterone | 80 | 25 |
| 2 | 271.2 | 253.1 | 40 | Dehydroepiandrosterone | 80 | 5 |
| 3 | 259 | 96.9 | 40 | Mannose 6-phosphate | 70 | 10 |
| 4 | 259 | 79 | 40 | Mannose 6-phosphate | 70 | 40 |
| 5 | 208.9 | 191.8 | 40 | Kynurenine | 80 | 5 |
| 6 | 208.9 | 93.9 | 40 | Kynurenine | 80 | 10 |
| 7 | 367.3 | 43.2 | 40 | Nervonic acid | 100 | 30 |
| 8 | 367.3 | 57.1 | 40 | Nervonic acid | 100 | 25 |
| 9 | 308 | 179 | 40 | Glutathione | 80 | 5 |
| 10 | 308 | 76 | 40 | Glutathione | 80 | 25 |
| 11 | 300.2 | 282 | 40 | Sphinganine | 70 | 5 |
| 12 | 300.2 | 55.1 | 40 | Sphinganine | 70 | 25 |
| 13 | 403.1 | 111 | 40 | Uridine−5'−diphosphate | 100 | 20 |
| 14 | 403.1 | 158.8 | 40 | Uridine−5'−diphosphate | 100 | 20 |
| 15 | 132 | 90 | 40 | Creatine | 100 | 10 |
| 16 | 132 | 44.2 | 40 | Creatine | 100 | 15 |
| 17 | 127 | 110 | 40 | 5-Aminoimidazole-4-carboxamide | 60 | 20 |
| 18 | 127 | 55.1 | 40 | 5-Aminoimidazole-4-carboxamide | 60 | 2 |

DP, declustering potential; CE, collision energy.

Supplementary Table 2 Linear regression equations, correlation coefficients, and linear ranges of the nine candidate metabolites.

| Candidate metabolites | Linear regression equations | correlation coefficients | linear ranges（µmol/L） |
| --- | --- | --- | --- |
| Dehydroepiandrosterone | y = 0.1897x - 0.0018 | 0.9941 | 0.005-0.5 |
| Mannose 6-phosphate | y = 0.0068 x + 0.0509 | 0.9973 | 1-500 |
| 5-Aminoimidazole-4-carboxamide | y = 0.0747 x + 0.1934 | 0.997 | 0.5-250 |
| Creatine | y = 0.0394x + 0.4712 | 0.9929 | 0.1-500 |
| Glutathione | y = 0.0096 x + - 0.0446 | 0.9989 | 1-500 |
| Kynurenine | y = 0.0378 x + 0.0004 | 0.9999 | 0.1-125 |
| Sphinganine | y = 0.3822 x + -0.634 | 0.9988 | 0.1-100 |
| Uridine−5'−diphosphate | y = 0.0031 x + 0.0097 | 0.9918 | 1-500 |
| Nervonic acid | y = 0.0004 x + 0.0011 | 0.9979 | 2.5-125 |
